# Organization of the Respiratory Supercomplexes in Cells with Defective Complex III: Structural Features and Metabolic Consequences

**DOI:** 10.3390/life11040351

**Published:** 2021-04-17

**Authors:** Michela Rugolo, Claudia Zanna, Anna Maria Ghelli

**Affiliations:** Department of Pharmacy and Biotechnology, University of Bologna, 40126 Bologna, Italy; michela.rugolo@unibo.it (M.R.); claudia.zanna@unibo.it (C.Z.)

**Keywords:** respiratory complexes, respiratory supercomplexes, oxidative stress, mitochondrial DNA, *MTCYB* mutations, cytochrome *b*, complex III, mitochondrial diseases

## Abstract

The mitochondrial respiratory chain encompasses four oligomeric enzymatic complexes (complex I, II, III and IV) which, together with the redox carrier ubiquinone and cytochrome *c*, catalyze electron transport coupled to proton extrusion from the inner membrane. The protonmotive force is utilized by complex V for ATP synthesis in the process of oxidative phosphorylation. Respiratory complexes are known to coexist in the membrane as single functional entities and as supramolecular aggregates or supercomplexes (SCs). Understanding the assembly features of SCs has relevant biomedical implications because defects in a single protein can derange the overall SC organization and compromise the energetic function, causing severe mitochondrial disorders. Here we describe in detail the main types of SCs, all characterized by the presence of complex III. We show that the genetic alterations that hinder the assembly of Complex III, not just the activity, cause a rearrangement of the architecture of the SC that can help to preserve a minimal energetic function. Finally, the major metabolic disturbances associated with severe SCs perturbation due to defective complex III are discussed along with interventions that may circumvent these deficiencies.

## 1. Introduction

The mitochondria are cytosolic organelles of eukaryotic cells in charge of ATP production through the process of oxidative phosphorylation (OXPHOS). However, several other important pathways are associated with mitochondria, such as the citric acid cycle [1,2], the fatty acids oxidation [3] and lipid droplets formation [4], the iron–sulfur (Fe–S) protein biogenesis [5] and amino acids catabolism [6]. Furthermore, mitochondria are implicated in the buffering of cytosolic calcium concentration [7], in generation of reactive oxygen species (ROS) [8], and in regulation and execution of different types of cell death [9]. They are also involved in an array of adaptive responses triggered by perturbations of intracellular homeostasis [10], orchestrating anabolic and catabolic reactions, which are finely adjusted according to different cytosolic conditions. All these interconnected functions are sustained by the activity of the “mitochondrial proteome”, estimated to contain at least 1000 (in yeast) [11] to 1500 (in humans) [12] different proteins, 15% of which are directly involved in energy metabolism and the OXPHOS system. Note that recent bioinformatics analysis in yeast provided evidence for more proteins than expected, cryptically localized inside mitochondria [13].

As typically described in the textbooks, mitochondria have two membranes, the outer membrane, which acts a barrier separating mitochondria from the cytoplasm, and the inner membrane surrounding the matrix, where soluble enzymes of intermediary metabolism, ribosomes and the mitochondrial genome (mtDNA) are hosted. The inner mitochondrial membrane is characterized by an extraordinarily high protein content and, in particular, encloses many copies of the respiratory chain components that together with the ATP synthase (named also complex V, CV) form the molecular machinery of OXPHOS, i.e., the ATP production from ADP and inorganic phosphate. The mitochondrial respiratory chain consists of four enzymatic multi-subunit complexes, namely the NADH-coenzyme Q reductase (Complex I, CI), the succinate-Coenzyme Q reductase (Complex II, CII), the ubiquinol-cytochrome *c* reductase (Complex III, CIII), and the cytochrome *c* oxidase (Complex IV, CIV). Two mobile redox-active compounds, the lipophilic coenzyme Q (CoQ) and the hydrophilic cytochrome *c,* connect the enzymatic complexes, thus allowing the electron transfer from soluble reducing equivalents (NADH and FADH_2_) to molecular oxygen.

Unlike the oxidation of NADH which only occurs via CI, FADH_2_ can be oxidized at the inner membrane mainly by CII, but also by other less abundant proteins such as the glycerol-3-phosphate dehydrogenase [14], the electron transfer flavoprotein dehydrogenases [15,16,17], the dihydroorotate dehydrogenase [18], the choline dehydrogenase [19], the sulfide CoQ reductase [20], and the proline dehydrogenase [21]. All these proteins are able to feed electrons to CoQ and in turn to CIII, which therefore can be considered the central collector delivering electrons through cytochrome *c* to CIV. The electron transport is coupled to proton extrusion from the matrix into the intermembrane space generating a transmembrane proton gradient at the level of CI, CIII and CIV, but not of CII. This later, together with other FAD-linked enzymes, does not contribute to energy conservation.

## 2. Mitochondrial Proteins Are Encoded by Two Genomes

Most mitochondrial proteins are encoded in the nucleus, synthesized in the cytosol and then imported into mitochondria by specific targeting mechanisms. However, these organelles are characterized by the presence of an independent genome, the mtDNA. This peculiarity is believed to be due to the evolutionary origin of mitochondria from alpha-proteobacteria integrated into proto-eukaryotic host, of which the details are still debated [22]. Most mitochondrial genes were then transferred to the nucleus, although a few of respiration-competent genes were conserved as an independent genome. The mtDNA is a circular double-stranded DNA, in humans of approximately 16.5 kb, encoding for thirteen polypeptides, all essential subunits of the OXPHOS system, and also for two ribosomal RNAs and twenty-two transfer RNAs, required for the intra-mitochondrial translation of the thirteen proteins. The evolutionary pathways involved in maintaining this transcriptionally active genome in addition to nuclear DNA are still poorly understood. New system biology and bioinformatics approaches have confirmed that the very high hydrophobicity of the proteins encoded by mtDNA is crucial to limit their translocation from the cytoplasm to the mitochondrial membrane and to favour mistargeting to the endoplasmic reticulum. In addition, the high CG content has been shown to increase the thermodynamic stability of the mtDNA, protecting from environmental insults [23]. Of note is that the high GC content might be related also to the surprisingly very high local temperature (about 50 °C) recently determined inside mitochondria [24]. Finally, the preferential encoding of components essential for organelle function in the mtDNA would allow localized control of gene expression and therefore the assembly of protein complexes [25].

## 3. Both Genomes Contribute to the Onset of Mitochondrial Diseases

Mitochondrial diseases are genetically heterogeneous disorders caused by mutations in nuclear genes encoding OXPHOS structural proteins or assembly factors, which are proteins required for the correct maturation of the complexes, but not contributing to the final structures. Mutations can also affect the molecular machineries of mtDNA replication and maintenance, of mitochondrial transcription and translation, as well as proteins involved in cristae shaping, network dynamics and quality control, composition of membrane lipids or mechanisms of antioxidant defences. Disorders can also be caused by mutations in the mtDNA, encoding structural OXPHOS subunits. In this case the genetic features are very peculiar, since the mtDNA inheritance mode follows the maternal lineage. Furthermore, each cell presents multiple copies (100–1000) of this genome, so that mutated and non-mutated copies can co-exist in the same individual, generating a phenomenon called heteroplasmy. Accordingly, the clinical phenotype and the severity of biochemical dysfunctions are highly variable, and pathology only manifests when the percentage of mutated mtDNA exceeds a threshold, which is variable for each kind of mutation. For a recent exhaustive review on the genetic basis of primary mitochondrial diseases we refer to Fernandez-Vizarra and Zeviani, 2020 [26].

## 4. The OXPHOS System

The structures of individual mitochondrial respiratory chain complexes have been determined by X-ray crystallography [27,28,29,30] or electron cryo-microscopy [31,32,33,34,35]. The bovine or human mitochondrial CI contains 44 different subunits, forming an L-shaped structure. The minimal functional unit of CI, which is conserved from bacteria to mammals, comprises 14 subunits known as core subunits. Subunits ND1-ND6 and ND4L form the hydrophobic membrane arm, the other seven core subunits form the hydrophilic arm protruding into the matrix and comprising a flavin mononucleotide (FMN) and eight iron–sulfur clusters as redox active prosthetic groups. This latter contains the NADH binding and electron transfer sites, whereas the membrane arm performs the proton translocation. The other supernumerary subunits play significant roles in the assembly, stabilization and regulation of CI [35,36,37].

CII is composed of four subunits, forming a hydrophilic head, containing a FAD binding protein and an iron–sulfur protein, and the hydrophobic arm with two membrane-anchor proteins (CybL and CybS). Three kinds of prosthetic groups, FAD, heme and iron–sulfur clusters, were recognized in CII, coupled with two Q-binding sites [38,39].

The mammalian CIII monomer is composed of three respiratory subunits (cytochrome *b*/MTCYB, cytochrome *c1*/CYC1 and the Rieske iron–sulfur protein/UQCRFS1, two core proteins (UQCRC1, UQCRC2) and six low-molecular-weight proteins (UQCRH/QCR6, UQCRB/QCR7, UQCRQ/QCR8, UQCR10/QCR9, UQCR11/QCR10 and a cleavage product of UQCRFS1). CIII is present as dimer (approx 450 kDa), although it is still controversial whether the two monomers of CIII_2_ function cooperatively or independently [40,41].

The CIV monomer has a mass of approximately 200 kDa and is believed to occur in the membrane both as a monomer and a dimer [42]. Each CIV monomer consists of 14 subunits [43], since NDUFA4, which was considered to be a subunit of CI, is actually a subunit of CIV [44]. The four redox-active metal centres constituting the electron transport pathway are heme *a3* and CuB, forming the binuclear centre that binds oxygen, and heme *a*, located in subunit COXI. The CuA center is incorporated in COXII [45]. The remaining subunits (COX4, 5A, 5B, 6A, 6B, 6C, 7A, 7B, 7C, 8A) are thought to have a structural role in the stabilization of the complex [46,47].

## 5. OXPHOS Optimization by the Inner Membrane Architecture

**Figure 1:**
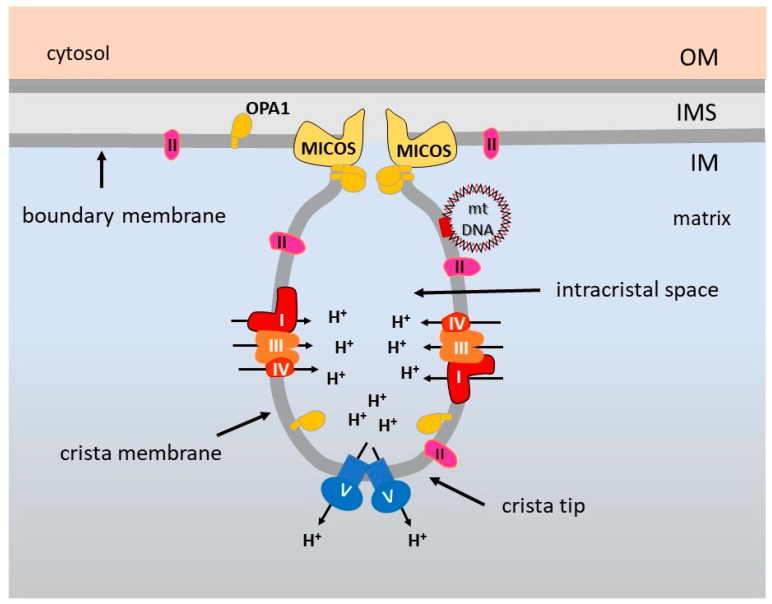
Architecture of the inner membrane and unequal distribution of OXPHOS complexes. CI, CIII and CIV are in the cristae membrane, CV dimers are at the cristae tip, whereas CII is also found in the inner boundary membrane. MICOS and OPA1 stabilize the cristae junctions, providing constrains to membrane mobility of complexes.

The general notion that the inner membrane is heavily folded to form the cristae has been reconsidered after 3D electron microscopy (EM) tomographic analysis in mitochondria from a wide variety of organisms, revealing that the cristae membrane opens toward the intermembrane space through narrow tubular structures, the crista junctions [48]. The molecular components of these cristae junctions have been subsequently identified, the most relevant being the mitochondrial contact site and cristae organizing system (MICOS) [49,50,51] and the high-molecular-weight GTPase Optic atrophy 1 (OPA1) [52,53,54] (Figure 1).

Furthermore, the observation of mitochondrial specimens in their native environment by means of the immuno-EM and cryo-EM tomography allowed to demonstrate that dimers of CV are arranged in long rows along the tightly curved ridges of the cristae membrane [55] (Figure 1). These ribbons of CV dimers are involved in the folding of the crista membrane [56]. It seems therefore that the intra-mitochondrial architecture is more complex than initially believed, with MICOS, OPA1 and CV dimers playing a structural role, together with the abundance of non-bilayer cone-shaped phospholipids, such as cardiolipin and phosphatidylethanolamine [53,57,58].

Of note is that the presence of MICOS and other proteins at the cristae junctions provides severe constraints to the mobility of protein complexes along the inner membrane, and therefore two sub-compartments with unequal protein distribution and functions can be envisaged: the cristae membrane, protruding in the matrix, and the inner boundary membrane, facing the outer membrane. The respiratory complexes are mainly found in the cristae membrane [56]. Further, sophisticated analysis of diffusion and directionality of movement of single complexes by super resolution microscopy revealed that all the complexes are trapped in the laminar part of cristae, except CII which is also found in the inner boundary membrane [59] (Figure 1). It has to be noticed that CII is a rather small complex and does not associate in supramolecular assemblies with other complexes. It follows that the specific geometry of the inner membrane, dictated by the cristae junction organization at the bottom and CV dimers on the top of crista, has a critical impact on the energetic function of mitochondria. As summarized in Figure 1, protons ejected by CI, CIII and CIV segregated on the laminar part of the cristae, are enriched in the intracristal space and flow back into the matrix through CV. This arrangement was predicted to be highly efficient in promoting energy conversion [56]. However, the intracristal membrane geometry has not been shown to influence the local pH gradients [60,61], consequently it has been proposed that the tight packing of the OXPHOS machinery in the cristae membranes favours the kinetic coupling between proton pumping and ATP synthesis [61]. The extraordinary power of super resolution live-cell imaging in combination with EM tomography and genome editing will further illuminate the functional details and dynamic aspects of this important microcompartment.

## 6. Supramolecular Organization of the Respiratory Complexes

The unequal protein complex distribution within the inner membrane subcompartments is also favoured by their size and their ability to interact with other complexes to form high-molecular-weight macromolecular aggregates. Differently form CII, which is always found alone, the proton-pumping CI, CIII and CIV can assemble in non-covalent associations defined as respiratory supercomplexes (SCs). Respiratory SCs were first identified by non-denaturing blue native gel electrophoresis (BN-PAGE) of mitochondrial membrane extracts, using the mild detergents Triton X-100 and digitonin, as high-molecular-weight gel bands, showing activity for CI, CIII and CIV [62,63]. The mammalian SCs containing CI, CIII dimer (CIII_2_), and CIV with different stoichiometry are sometimes referred to as respirasomes, because they contain all the components required to transfer electrons from NADH to molecular oxygen [63,64,65]. In human cells, the respirasome comprises most of CI (>90%), approximately 40–50% of CIII_2_ and 20–30% of CIV. Differently from CI, significant amounts of CIII_2_ (50–60%) and CIV (70–80%) can be also found as isolated complexes within the membrane. The CIII+CIV SC represents about 5% of total amount of the complexes [66].

### 6.1. The CI+CIII_2_+CIV SC or Respirasome

The structures of the CI+CIII_2_+CIV SC isolated from different mammalian mitochondria have been determined by single-particle electron cryo-EM at resolutions ranging from ~30 to ~4 Å [67,68,69,70] or by electron cryotomography (cryo-ET) at ~30 Å resolution [71,72].

**Figure 2:**
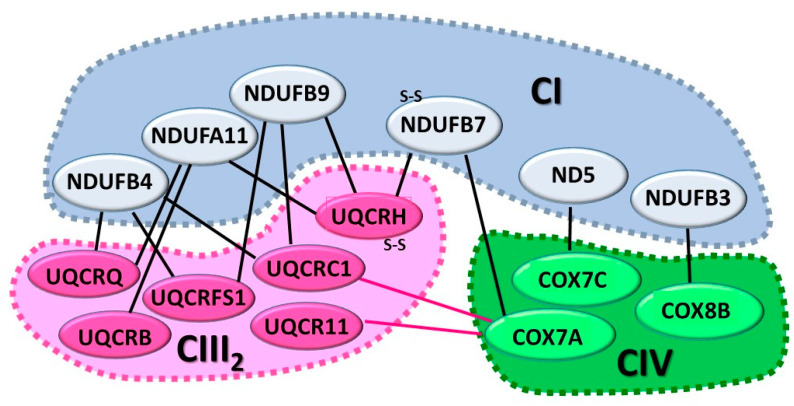
Proposed interactions between the respiratory complexes of the respirasome (according to [69]). Nomenclature of human subunits is indicated. The number of tight contacts between CI and CIII is greater than those between CI–CIV and CIII–CIV.

In the structure of respirasome, CIII_2_ borders the concave arc of CI membrane arm, and CIV is located near CIII_2_ at the distal end of the CI membrane arm, with cardiolipin molecules filling the gaps between the individual complexes [67,71]. In the respirasome, two distinct arrangements have been identified, a major “tight” and a minor “loose” form, which mainly diverge for the position of CIV. As illustrated in the cartoon of Figure 2, the most extensive and stable interactions take place between CI and CIII_2_.

Two are the major interaction points: in the inner membrane between CI subunit NDUFA11 and the CIII subunits UQCRB, UQCRQ and UQCRH, and at the matrix between the CI subunits NDUFB4 and NDUFB9 and CIII subunits UQCRC1 and UQCRFS1 [69]. Another important interaction occurs between CI subunit NDUFB7 and subunit UQCRH on CIII_2_. Of note is that both subunits contain disulphide bonds, suggesting that redox regulation might modulate the interactions between the respiratory complexes [69].

Few interactions occur between CI and CIV, the most important linking the CI ND5 subunit to COX7C at the interface between matrix and inner membrane, the other between NDUFB3 subunit and COX8B. The contacts between CIII and CIV mainly involve the CIV COX7A subunit with the UQCRC1 and UQCR11 subunits and COX5B subunits with the UQCRC1 subunit [69].

Interestingly, high-molecular-weight bands above the CI+CIII_2_+CIV SC were previously described by BN-PAGE analysis [63]. More recently, mass spectrometry analysis has suggested that the main components of these bands are subunits of CI, CIII and CIV, and EM analysis detected a minor population of particles with circular arrangements. This led to proposing a higher oligomeric state named megacomplex CI_2_+CIII_2_+CIV_2_. This assembled structure is shaped by a central CIII_2_ surrounded by two copies each of CI and CIV. This arrangement may be an oligomerization form of respiratory complexes operating under the most efficient emergency conditions, because both monomers of the CIII dimer could receive CoQH_2_ from each CI and pass reduced cytochrome *c* to one adjacent CIV. Further analysis by cryo-EM allowed to better define the architecture of the megacomplex [73], although these results were intensely debated, mainly due to limitations of cryo-ET technology in the reconstruction of supramolecular assemblies.

### 6.2. The CI+CIII_2_ SC

CI can also assemble with the CIII dimer alone to form the CI+CIII_2_ SC. Recently, a functional CI+CIII_2_ SC has been isolated from ovine heart mitochondria and characterized by cryoEM, demonstrating that the contacts between CI and CIII_2_ are evolutionarily conserved [72] and are similar to those of the respirasome, confirming the stabilizing role of CIII_2_ on CI [69,74].

### 6.3. The CIII_2_+CIV SC

Isolated CIII_2_ and CIV coexists with the CIII_2_+CIV SC, which has not been structurally characterized in mammalian tissues, likely due to its low relative abundance. Cryo-EM studies on yeast, which lacks CI, detected a CIII dimer at the core of the SC flanked by a CIV monomer on either side. The CIII–CIV interface revealed protein-protein interactions on either side of the membrane and with lipids within the membrane. The majority of interactions occur on the matrix side between Cor1/UQCRC1 and the N-terminus of COX5A, whereas the C-terminal domain of COX5A interacts with both Qcr6/UQCRH and cytochrome *c*1 on the intermembrane space. Within the membrane, COX5A contacts the N-terminal helix of Rip1/UQCRH and Qcr8/UQCRQ via a cardiolipin molecule and another lipid modelled as phosphocholine. Two other cardiolipins indirectly support the CIII–CIV interface highlighting again their crucial role in SC formation [75].

## 7. SCs Assembly Factors

Several molecules able to directly or indirectly promote mitochondrial SCs assembly and stability have been identified, such as cardiolipins, stomatin-like protein 2, prohibitin 1 and 2, and others (for a recent review, see [76]). Here we consider the protein factors involved in SCs assembly, the most studied being HIGD2A (hypoxia inducible domain family member 2A) [66,77,78,79,80] and COX7A2L [81]. HIGD2A mainly functions in CIV assembly [77,82] and mediates CIV integration within the respirasome, as well [66,80]. The mammalian homologous HIGD1A has been reported to assist the assembly of CIII and CIII-containing SCs biogenesis, having overlapping functions with HIGD2A [82]. These findings suggest the involvement of multiple pathways to assemble the respiratory complexes and to gather the SCs [82,83,84].

COX7A2L (also called SCAF1) has been the subject of various studies with contradictory results, due to the presence of two variants (long and short forms) differently expressed in mouse strains and tissues [81]. There is an agreement that long COX7A2L can bind to both CIII_2_ and CIV and is required for formation of the CIII_2_+IV SC [79,83,84], as well as for the assembly of the megacomplexes [73], but is dispensable for the respirasome [66,80].

Assembly factors specifically interacting with CIII were identified in yeast and their orthologs validated in human cells, namely, Cbp3 (UQCC1) and Cbp6 (UQCC2) [85] and Cbp4 (UQCC3) [86]. The best-characterized is UQCC3 (ubiquinol-cytochrome *c* reductase complex assembly factor 3; also known as C11orf83), which is involved in the early phase of CIII assembly and in the stabilization of CIII-containing SCs [86,87]. Interestingly, UQCC3 was reported to be indispensable for simultaneously maintaining both OXPHOS and glycolysis during hepatocarcinoma cells hypoxia adaption, suggesting a role in energetic reprogramming [88].

Table 1 summarizes the genetic alterations affecting CIII structural subunits and assembly factors, which will be considered in the following paragraphs, also indicating the redox activities and the assembly state of complexes/SCs.

## 8. Models of SC Organization

The question of the organization of the respiratory chain has been debated since the pioneering studies aimed at identifying the molecular components and catalytic mechanisms [89,90]. Hackenbrock et al. (1986) described the ”fluid” or “random collision model” of electron transfer, where each complex acts as an individual entity, CoQ and cytochrome *c* freely diffuse within the lipid bilayer, and electron transfer occurs during random and transient collision events [91]. The alternative solid-state model derives from the early observations reporting that CI and CIII preferentially associated in the native membranes [92]. Since 2000, the solid-state model has received strong support from isolation of SCs by BN-PAGE and then by development of protein crystallography and cryo-EM approaches. According to the solid model, a unique multicomplex unit is able to execute all the steps of respiration. More recently, the two models have been merged in the so-called “plasticity” model [64,93], based on the coexistence of individual CIII and CIV complexes, with a variable combination of SCs. Dynamic SC association/dissociation can be triggered under physiological conditions by availability of different substrates (NADH and FADH_2_), determining a variety of different structural options that allow to adapt the efficiency of the respiratory chain to metabolic demands. One consequence of the possibility to preferentially use NADH- or FADH_2_-dependent substrates through isolated complexes and SCs is the presence of partially dedicated pools of the mobile electron carrier CoQ [81,94]. However, demonstrating the presence of two CoQ pools is experimentally difficult and therefore this issue has been disputed [95,96]. According to the working model proposed by the Enriquez group, under normal circumstances, the superassembly in the respirasome generates a CoQ fraction within the SCs functionally dedicated to NADH oxidation. Given that also individual CIII_2_ co-exist with the SC, CoQH_2_ generated by CII or by other FADH_2_-dependent enzymes can be oxidized by the free CIII out of respirasome. Under conditions of block/lack of CIII or CIV, all CIII is associated with CI and CoQH_2_ can diffuse out and be oxidized outside the SCs, and, on the other hand, the ubiquinol generated by CII can diffuse in and be oxidized by CI+CIII_2_ SC [81,94]. Additionally, for cytochrome *c* the existence of a unique pool is also unlikely, given that this protein, besides transferring electrons between CIII and IV, can interact with many mitochondrial and non-mitochondrial components, exerting a variety of roles, among them the trigger of apoptotic cell death. SCAF1, which has been shown to be required for CIII and CIV interaction, plays an important role in the cytochrome *c* pool functional segmentation and likely in the efficient use of respiratory substrates. For a detailed discussion on CoQ and cytochrome *c* segmentation in SCs, we refer to [97].

## 9. Functional Roles of the SCs and CIII Involvement

Even if the evidence in support of SCs in detergent extracts is beyond question and the SCs in situ arrangement in the mitochondrial inner membrane has been defined, their physiological functional significance is still debated. The hypothesis of a catalytic advantage provided by SCs [67,81,98] has been questioned [65,72,95,96,99]. Catalytic advantage would imply substrate channelling, i.e., a defined conduit for the hydrophobic quinol from its reduction site in CI to CIII, where it is oxidized. However, no structural evidence for such a protein-defined conduit between CI and CIII_2_ was obtained [100].

As alternative hypotheses, it has been proposed that SC organization could support CI assembly and stability [101,102], preventing dangerous casual protein aggregations within the membrane [100,103]. Finally, it has to be recalled that CI and CIII_2_ are the main sources of reactive oxygen species (ROS) in the mitochondrial matrix and in the inner membrane [104,105,106,107], and that experimental dissociation of the SCs results in increased ROS production from CI [108]. It was, therefore, proposed that SCs organization could reduce ROS generation and subsequent oxidative damage to membrane components [108,109], although the molecular mechanism was not clearly defined. Analysis of recent structural data allowed to propose that in the respirasome, the interactions of CIII_2_ with CI and CIV break the symmetry of CIII_2_, ensuring efficient oxidation of QH_2_ and allowing CI to operate at full rate, thus helping to reduce ROS formation at CI [100].

The relationship between SCs and ROS is intriguing, because it is difficult to dissect how important SCs are to prevent ROS production and how ROS production depends on SCs disassembly. There is evidence that the production of ROS due to CIII dysfunction plays a role in the stability of SCs. It has been reported that some pathogenic mutations in *BCS1L*, the chaperone protein needed to incorporate the UQCFRS1 protein in CIII, affected CIII activity, inducing ROS production and secondary CI and CIV activity/stability alteration [110]. Furthermore, in a mouse cellular model lacking UQCFRS1 protein, the increase of ROS production due to CIII deficit induced a general reduction in the assembly and stability of CI, CIV, and in turn of SCs; however, this latter defect was partially rescued in presence of antioxidants treatment or hypoxia [111].

In addition to a direct ROS production by CIII due to its defective activity, it has been shown that the lack of CIII, by increasing the CoQH_2_/CoQ ratio, could promote the backflow of electrons from CoQH_2_ to CI by the reverse electron transfer (RET) reaction through CI. RET produced the oxidation of cysteine residues of CI, triggering its degradation and in turn hampering SC formation [94]. Although in this study the occurrence of RET and ROS production was not directly demonstrated, the ROS involvement was suggested by partial rescue of CI assembly after treatment with the CI inhibitor rotenone and under hypoxic conditions, all interventions that prevent RET. The overexpression of SOD2, however, was not effective [94]. An interesting finding has been recently reported, showing that Na^+^ modulates ROS production during acute hypoxia through the regulation of inner membrane fluidity [112]. Noticeably, this increased ROS production was associated with reduction in combined CII+CIII enzymatic activity and respiratory capacity, whereas combined CI+CIII activity and respiration remained unchanged. This finding reinforces the general idea of that formation of the CI+CIII_2_ SC limits the production of ROS.

Finally, in a human cellular model carrying an 18-bp frame deletion in *MTCYB* associated with a severe impairment of CIII, CI and CIV assembly and ROS production, treatment with the antioxidant N-acetyl cysteine partially rescued respirasome formation [113]. Taken together, these data indicate that ROS production may affect respiratory complexes and SCs assembly, thus, it is reasonable to speculate that the correct assembly of SCs could be useful to reduce ROS production in a healthy respiratory chain.

The fact that the mutual arrangement of CI and CIII_2_ is essentially conserved from obligate aerobic yeast to mammals, and plants seems to favour the hypothesis that specifically these two complexes have a functional role in maintaining the respiratory chain stability reducing ROS production [51]. Further structural and biochemical work is needed, also considering that in some cases a slight increase of ROS was detected, but the assembly of respiratory complexes and SCs was normal, suggesting that ROS levels could modulate the response to this supermolecular organization [114,115,116]. The influence of tissue-specific subunit isoforms is also to be taken into account [83].

## 10. SCs Biogenesis and Role of CIII

An important issue to be elucidated concerns the SCs biogenesis, i.e., how CI, CIII and CIV interact in the respirasome formation. Currently, two models have been presented. The first model proposes that the close interplay among the three complexes favours better structural stability, implying that the isolated forms of respiratory complexes, in particular CI, are more prone to degradation. Accordingly, the complexes are supposed to follow separate assembly pathways to build mature individual complexes and to form SCs in a second step [93,117]. The second model suggests a central role of CI acting as a scaffold for the sequential incorporation of CIII_2_ and CIV_n_ subassemblies to form mature SCs [102]. However, this latter model does not rule out the occurrence of a dynamic exchange of CIII_2_ and CIV once the respirasome assembly has been completed, allowing the formation of the other SCs and the co-existence with free complexes [66]. Both models have been developed from data obtained in experiments in which SCs biogenesis was studied following the time-course of respiratory complexes assembly after mitochondrial translation inhibition, by using diverse antibiotics and different experimental conditions, and this may explain some conflicting results. Recently, interesting information obtained by combining the Stable Isotope Labelling by Amino acids in Cell culture (SILAC) and complexome profiling techniques, suggested a new main role of CIII in SCs formation as a structural and functional platform for the overall respiratory chain biogenesis [118,119]. In particular, Protasoni et al., 2020 [118] analyzed human cells bearing a 4-bp *MTCYB* deletion that induced a frameshift with the loss of the encoded protein and of CIII_2_, associated with hampered CI biogenesis due to the stall in N-module incorporation and decreased CI stability. Furthermore, the CIV assembly was also defective because some CIV subunits were recruited within the accumulated CIII subassemblies. A similar analysis was carried out by Páleníková et al. (2021) [119], in cells bearing a 18-bp frame deletion in *MTCYB* gene that produced a protein shortened of six amino acids and induced a strong defect of CIII_2_ activity/assembly as well as of CI and CIV [113,120,121]. These cells, differently from those with the 4-bp deletion, exhibited some amount of isolated CIII_2_ and CI, that were associated in the enzymatically active CI+III_2_ SC [113,121], in agreement with structural data demonstrating the existence of multiple interactions between the two complexes [74]. However, Páleníková et al. (2021) showed that several sub-complexes with mixed CIII, CI and CIV subunits were also present, indicating that the decreased CIII_2_ assembly leads the formation of intermediates that trap other respiratory complex subunits impairing their assembly [119]. Taken together, these data support a cooperative assembly model in the respiratory chain structural and functional maturation and in SCs biogenesis, highlighting the central role of CIII_2_ as scaffold for the ordered association with mixed CI and CIV subunits. Exhaustive studies focused on the mechanisms of SCs biogenesis in presence of CIII deficiency are still lacking; however, after reviewing literature it appears that mutations that disrupt CIII assembly also induce an impairment of CI and CIV and of SCs formation as well. Indeed, severe mutations in both CIII structural subunits or in early assembly factors strongly affect CIII structure and reduce CI and CIV stability [85,86,111,112,118,122,123,124,125,126,127,128] (Table 1). Unfortunately, several reported mutations that could be relevant for CIII structure, such as truncating mutations in cytochrome *b*, were poorly or not at all investigated for CIII assembly and related influence on CI, CIV and SCs structure (Table 1). However, it appears that most *MTCYB* missense mutations affect neither the assembly of CIII nor CI and CIV (Table 1), except for the p.Y278C mutation that was associated with a slight reduction of CI activity only and a reduced amount of the CIII_2_ +CIV SC [129], and the p.E373K that disassembled CIII and CI, but not CIV [130]. Taken together, these findings indicate that the assembly of CI and CIV depends on the physical presence of assembled CIII species and not on their catalytic activity, although ROS production could play a role in specific cases [111]. However, the role of CIII structure in SCs biogenesis is far from being clarified; for instance, some authors suggested that the complete formation of the CIII_2_+CIV SC is important to safely activate CI only when the respiratory chain is fully assembled [102], but some data show that CI could interact with a pre-CIII to form enough SC to ensure sufficient respiratory chain activity [115,131]. These data suggest that regardless of the mutation, there is a tendency to maintain respiratory complex stability and SC assembly to mitigate CIII dysfunction. This latter piece of evidence is supported by recent papers showing that missense mutations in *MTCYB* that induce defective CIII enzymatic activity when detected in the isolated complex, are mitigated when CIII activity is measured under conditions in which the respiratory complex is organized into SCs [129,132]. Although further work is clearly needed, recent structural details obtained from cryo-EM analysis of active SC particles from sheep mitochondria highlighted the specific involvement of cytochrome *b* in the crosstalk between CI and CIII_2_, confirming its role in structural/functional interactions between the two complexes [74].

## 11. Metabolic Disturbances and Treatment Options

**Figure 3:**
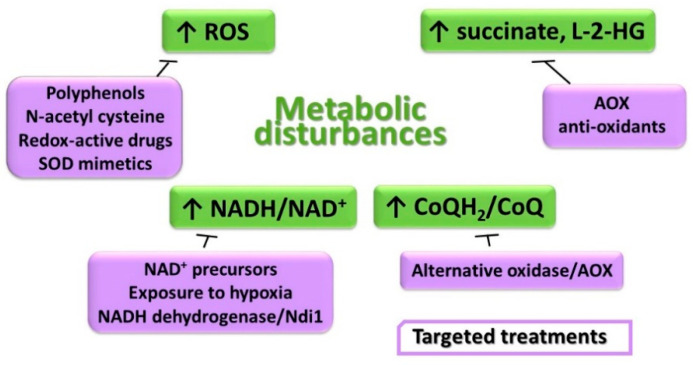
Major metabolic alterations associated with perturbed supramolecular organization of CIII-containing SCs and proposed treatments.

Defective CIII and associated perturbation of the supramolecular organization of CIII-containing SCs result in several significant metabolic alterations, which are briefly summarized in Figure 3 and discussed in detail below, providing also some hints on experimental treatments specifically aimed at improving these metabolic disturbances. For an extensive recent review of the therapeutic strategies to treat mitochondrial disease, we refer to [133].

### 11.1. Unbalanced Intracellular Redox Homeostasis

It is widely accepted that even under physiological conditions the electron flow through the mitochondrial respiratory chain results in mild ROS production [134,135], with CI and CIII being the main redox components responsible for molecular oxygen reduction to superoxide anions [104,105,106,107,136]. As mentioned above (point 9), one of the proposed roles of the supramolecular organization into SCs is to limit ROS formation, avoiding the diffusion of free radicals with damaging effects at protein and lipid levels [100,137,138]. Accordingly, different experimental conditions causing disruption or prevention of the association between CI and CIII were shown to increase ROS production, supporting the view that dissociation of SCs may strictly link oxidative stress and energy failure [108]. Furthermore, pathological conditions leading to dismantling the SCs organization, such those described in cells with 4- and 18-bp *MTCYB* deletions, were also shown to enhance ROS generation [94], and, due to up-regulation of intracellular efficient antioxidant defences in both cytosol and mitochondrial compartments, to cause a significant unbalance in the redox homeostasis [113,121,139]. Of note, mild to moderate increase in oxidative stress associated with parallel SCs depletion was detected in different brain areas of neuron-specific mice KO for the UQCRFS1 gene, demonstrating that ROS can modulate the SCs architecture to cope with a high level of ROS [140]. This conclusion is further supported by previous finding that a superoxide dismutase mimetic compound and SOD2 overexpression induced a partial increase in SCs in the UQCRFS1 KO cells [111,140]. Moreover, the prolonged treatment of cells bearing the 18-bp *MTCYB* deletion with N-acetyl cysteine (NAC) significantly increased the rate of ATP synthesis driven by CI substrates as well as the amount of free CI, CIII and CIV and of the respirasome. It is likely that NAC may provide optimal redox conditions for respiratory complexes interactions and SCs re-organization [113], also considering that both CI subunit NDUFB7 and CIII subunit UQCRH contain disulphide bonds [69].

### 11.2. Accumulation of the Reduced Form of Pyridine Nucleotides and CoQ

Direct consequences of defective CIII are energy failure and metabolic derangements, as indicated by a huge number of case reports describing lactic acidosis and hypoglycaemia as recurrent clinical phenotypes of patients bearing mutations in different CIII-related genes, i.e., *MTCYB* [120,141], *UQCRC2* [127,142], *UQCC3* [86] and others. Metabolomics analyses in liver of the mouse model of CIII dysfunction (*Bcs1l^c.232A>G^* mutant) revealed a decrease in carbohydrate intermediates, demonstrating an increase in glycolysis to compensate for the reduced mitochondrial ATP production [143]. Subsequently, targeted metabolomics detected increases in glucogenic and ketogenic amino acids in circulation, supporting a starvation-like condition [144]. Of note is that this mouse model, despite the severe CIII dysfunction, does not present significant perturbations in the SCs organization [115].

On the other hand, when the assembly of CIII-containing SCs is compromised, in addition to CIII, the amount of CI collapses as well, leading to elevation of the cellular ratio of reduced and oxidized pyridine nucleotides (NADH/NAD^+^). The inability to oxidize NADH in the mitochondrial matrix affects not only the efficiency of OXPHOS but also the flux of metabolites through the Krebs cycle. As a consequence, the cells become heavily dependent on aerobic glycolysis for survival. The glycolytic flux relies on the activity of glyceraldehyde-3-phosphate dehydrogenase which requires NAD^+^, generated from NADH oxidation by the cytosolic lactate dehydrogenase enzyme. In agreement with this notion, we found that the amount of lactate released into the growth medium by the homoplasmic cells bearing the 18-bp *MTCYB* deletion was significantly greater than WT cells. Noticeably, cells bearing the p.278Y>C *MTCYB* mutation impairing CIII activity without affecting SCs organization failed to increase lactate release [121], in accord with the clinical phenotype of patients, presenting lactic acidosis in the patient bearing the 18-bp *MTCYB* deletion [120], but not in that with the p.278Y>C mutation [145]. The molecular mechanism underlining this metabolic switch is unknown, although the possible role for UQCC3 may be worth investigating [88].

Interestingly, previous studies described benefits in lifespan and energetic function of defective CI by interventions targeting NADH elevation, such as supplementation with NAD-precursor [146] inhibition of mTOR [147] and of mitochondrial serine catabolism [148], as well as hypoxia treatment [149]. To circumvent the CI deficiency, some studies took advantage of the xenotopic expression of the single-subunit yeast enzyme NADH dehydrogenase (Ndi1) [150,151,152]. In yeast, Ndi1 catalyses the oxidation of NADH in the matrix like CI, but is unable to restore the proton pumping. Ndi1 protein expression in human cultured cells lacking CI restored the NADH-dependent respiration as well as the growth in glucose-free medium containing galactose [153,154]. Recently, McEllroy et al. (2020) generated a mouse that conditionally expresses Ndi1, confirming that its expression dramatically prolong lifespan, but was unable to significantly improve motor function in a mouse model of Leigh syndrome due to loss of the NDUFS4 CI subunit [155]. In the absence of structural data showing Ndi1 association with SCs, it is reasonable to speculate that the ability of Ndi1 to ameliorate the cell viability does not depend on association with other respiratory complexes, rather it depends on the restoration of NADH oxidation allowing for a compensatory increase in glycolysis and sufficient metabolite flux in the Krebs cycle.

At cellular level, the primary consequence of the specific drop/lack of CIII is the blockade of CoQH_2_ oxidation, preventing the NADH and FADH_2_ oxidation by CI and CII. As mentioned above, elevation of CoQH_2_/CoQ ratio causes reverse electron transport through CI, with local generation of superoxide, triggering CI subunits degradation and tuning the amount of this complex [94]. This is in agreement with previous data showing that CIII can be released from CI-containing SCs under metabolic conditions (e.g., starvation) when electron flux from FAD overwhelms the oxidation of CoQ, supporting the plasticity model of SCs organization [81]. The alternative oxidase (AOX) is a single-protein electron transport system present in bacteria, lower eukaryotes and plants that can perform CoQH_2_ oxidation instead of CIII and CIV, by transferring electrons directly from quinols to oxygen without proton translocation [156]. The xenotopic expression of tunicated AOX in mouse was recently investigated, failing to show any association of AOX with SCs. This finding supports the notion that xenotopically expressed AOX acts as a freely diffusible redox partner [157]. Of note is that the expression of AOX from *Emericella nidulans* in *MTCYB* KO cells induced CoQH_2_ oxidation, thus reducing the oxidative stress and inhibiting CI degradation. Despite the increased CI amount, the SCs were not restored due to the lack of CIII [94], further corroborating the central role of CIII as a scaffold for incorporation of CI and CIV [118]. Furthermore, AOX was reported to provide a full functional rescue of the cardiomyopathy of the *Bcs1l*^c.232A>G^ mutant mice, by restoring respiration to wild-type level. Noticeably, the CIII and CI+CIII_2_+CIV assembly was partially rescued in cardiac mitochondria, likely secondary to the general improvement in mitochondrial structure and function [144].

The observation that expression of a single enzyme, such as AOX, can bypass defective oxidative reactions carried out by dozens of proteins is intriguing. Besides representing a useful tool for detecting the contribution of ATP requirement from the NAD^+^ and CoQ regeneration, the oxidase may be of potential use for respiratory chain deficiencies, although a gene therapy approach seems quite problematic at present. In fact, it has to be considered that correction of the mutated gene in affected tissue of monogenic diseases by CRISPR/CAS9 genome editing, already shown to be promising in animal models, requires sophisticated gene-specific tools and is still under development for mtDNA interventions. Conversely, the expression of one protein such AOX might be, in theory, beneficial for restoring most of the metabolic stress induced by OXPHOS impairment caused by a wide variety of mutations.

### 11.3. Elevation of Succinate and Effects on Gene Expression Regulation

The lack of CIII and the extremely limited availability of oxidized CoQ results in the inability of the Krebs cycle to progress from succinate to fumarate, as demonstrated by the markedly increased levels of succinate and reduction of fumarate and malate detected by us in cells with the 18-bp *MTCYB* deletion [121] and also in cells bearing the 4-bp *MTCYB* deletion [158]. In cells with the p.278Y>C *MTCYB* mutation, with normal SCs organization, we also detected a weak increase of succinate, but increased malate and normal fumarate, suggesting that some succinate can be oxidized to fumarate which in turn produces malate, likely as a consequence of CIII assembly in the respirasome, that can preserve significant electron transport [121]. Elevation of succinate and fumarate was reported in the liver of *Bcs1l^c.232A>G^* mutant mouse, in line with the blockade of the Krebs cycle flux [159].

Succinate accumulation in the cytosol has been shown to have a strong impact in gene expression regulation, by inhibiting the 2-oxoglutarate-dependent dioxygenases, which catalyse hydroxylation reactions on various types of substrates. In particular, succinate, competing with 2-oxoglutarate, inhibits the activity of prolyl hydroxylases, leading to stabilization of Hypoxia Inducible Factor-1α (HIF-1α) under normoxia, defined as a pseudo-hypoxic condition [160]. HIF-1α can then translocate from cytoplasm to the nucleus where it associates with HIF-1β, to activate transcription of HIF-1α-target genes, among which are those encoding glycolytic enzymes [161]. It is likely that the huge increase of succinate levels determined in cells with the 18-bp *MTCYB* deletion may be at least in part responsible for the glycolytic switch revealed by increased extracellular lactate production [121].

In addition to succinate and fumarate, the levels of the oncometabolite 2-hydroxyglutarate (L-2-HG) were increased in RISP/ UQCRFS1 KO cells [162] and also in cells bearing the 4-bp *MTCYB* deletion [158]. All these Krebs cycle metabolites competitively inhibit the activity of 2-oxoglutarate-dependent dioxygenases, including also JmJC domain-containing histone lysine demethylases and ten-eleven translocation TETs family of 5-methlycytosine hydroxylases, involved in oxidizing 5-methylcytosine into 5-hydroxymethylcytosine [163]. Accordingly, the DNA and histone methylation were increased upon loss of RISP in fetal hematopoietic stem cells, impairing their differentiation and maintenance of stemness [162]. The increase in these metabolites, through inhibition of the histone and DNA demethylases, can therefore represent a very important factor affecting the epigenetic landscape of the cells and causing wide-ranging effects on cell physiology (for a review on Krebs metabolites and epigenetics, see [164]).

## 12. Conclusions and Perspectives

The development of cryo-EM technology has provided a powerful tool to analyse at atomic level the specific associations of CI, CIII and CIV into the respirasome, the CI+CIII_2_ and the CIII_2_+CIV_1-2_ SCs. However, these techniques suffer from some limitations, mainly associated with the mitochondrial purification procedures and the type/amount of detergent. In this regard, the development of the “in situ” reconstruction of SCs in eukaryotic cells in vivo by using the proximity-dependent labeling followed by mass spectrometry will open the possibility to identify potentially interacting proteins and their subcellular spatial localization [165,166]. Dynamic rearrangements between individual complexes and SCs have been demonstrated to occur (Table 1). It is necessary to identify the functional consequences of these arrangements as well as their implications in the regulation of the respiratory function under different physiological conditions. More biochemical and biophysical experiments, in combination with advances in super-resolution light microscopy are needed for clarify the functional mechanism of the SCs. This information will be crucial to elucidate the pathogenic mechanisms underlying the mitochondrial disorders associated with both nuclear and mtDNA mutations, and, hopefully, to identify effective treatments.

## Figures and Tables

**Table 1:** Disease genes encoding structural subunits and assembly factors associated with CIII deficiency.

Mutated Gene	Mutation	Enzymatic Activity			Isolated Complexes Assembly			Supercomplexes Assembly			Refs.
	CIII	CIII	CI	CIV	CIII	CI	CIV	CI+CIII+CIV	CI+CIII	CIII+CIV	
	**Structural subunits**										
***MTCYB***	p.14I> *	↓	↓	↓	↓	↓	↓	↓	↓	↓	[118,167,168]
	p.34G>S	↓	mild ↓	=	n.d.	n.d.	n.d.	n.d.	n.d.	n.d.	[169]
	p.35S>P	↓	n.d.	n.d	n.d.	n.d.	n.d.	n.d.	n.d.	n.d.	[170]
	p.40C>R	mild ↓	mild ↓	mild ↓	n.d.	n.d.	n.d.	n.d.	n.d.	n.d.	[171]
	p.113W> *	↓	n.d.	n.d	n.d.	n.d.	n.d.	n.d.	n.d.	n.d.	[169]
	p.135W> *	↓	n.d.	n-d	↓	n.d.	n.d.	n.d.	n.d.	n.d.	[172]
	p.141W> *	↓	=	=	n.d.	n.d.	n.d.	n.d.	n.d.	n.d.	[169]
	p.142G> *	↓	↓	n.d.	n.d.	n.d.	n.d.	n.d.	n.d.	n.d.	[173]
	p.151S>P	↓	n.d.	n.d.	↓	n.d.	n.d.	n.d.	n.d.	n.d.	[172]
	p.166G> *	↓	n.d.	n.d.	n.d.	n.d.	n.d.	n.d.	n.d.	n.d.	[174]
	p.166G>E	↓	n.d.	n.d.	n.d.	n.d.	n.d.	n.d.	n.d.	n.d.	[175]
	p.∆251-258	↓	mild ↓	=	n.d.	n.d.	n.d.	n.d.	n.d.	n.d.	[169]
	p.271E>K	mild ↓	=	=	=	=	=	=	=	=	[132]
	p.278Y>C	↓	mild ↓	n.d.	=	=	=	mild↑	mild↑	↓	[129,145]
	p.290G>D	↓	n.d.	n.d	n.d.	n.d.	n.d.	n.d.	n.d.	n.d.	[176,177]
	p.297S>P	↓	=	=	↓	=	=	n.d.	n.d.	n.d.	[178]
	p.∆300-305	↓	↓	↓	↓	↓	↓	↓	↓	↓	[113,120]
	p.318K>P	↓	↓	=	↓	↓	=	n.d.	n.d.	n.d.	[122]
	p.326W> *	↓	n.d.	n.d	n.d.	n.d.	n.d.	n.d.	n.d.	n.d.	[169]
	p.339G> *	↓	n.d.	n.d	n.d.	n.d.	n.d.	n.d.	n.d.	n.d.	[179]
	p.339G>E	↓	n.d.	n.d	n.d.	n.d.	n.d.	n.d.	n.d.	n.d.	[180]
	p.352Q> *	↓	↓	↓	n.d.	n.d.	n.d.	n.d.	n.d.	n.d.	[123]
	p.373E>K	↓	↓	=	↓	↓	=	n.d.	n.d.	n.d.	[130]
***UQCRB***	Change at C-term	↓	↓	↓	n.d.	n.d.	n.d.	n.d.	n.d.	n.d.	[124]
***UQCRQ***	p.45S>F; p.45S>F	↓	↓	=	n.d.	n.d.	n.d.	n.d.	n.d.	n.d.	[125]
***CYC1***	p.96W>C; p.215L>F	↓	↓	↓	n.d.	n.d.	n.d.	n.d.	n.d.	n.d.	[126]
***UQCRC2***	p.183R>W; p.183R>W	↓	↑	↓	↓	↓	=	↓	↓	n.d.	[127]
	p.183R>W; p183R>W	↓	↓	=	↓	↓	n.d.	n.d.	n.d.	n.d.	[142]
***UQCRFS1***	p.14V>D; p.204R> *	reduced overall respiration			↓	n.d.	n.d.	n.d.	n.d.	n.d.	[181]
	p.72V>T81del10; p.72V>T81del10	reduced overall respiration			↓	n.d.	n.d.	n.d.	n.d.	n.d.	[181]
	mouse KO	↓	↓	↓	↓	↓	↓	↓	↓	↓	[111]
	**Assembly Factors**										
***BCS1L***	p.35G>R; p.184R>C	↓	n.d.	n.d.	↓	n.d.	n.d.	=	n.d.	↓	[182]
	p.45R>C; p.56R> *	↓	↓	=	↓	↓	↓	n.d.	n.d.	n.d.	[110,183]
	p.50T>A; p.50T>A	mild ↓	n.d.	n.d.	mild↓	=	n.d.	n.d.	n.d.	n.d.	[110,184]
	p.R56 *; g1181A>G/g1164C>C	↓	=	↓	↓	=	↓	n.d.	n.d.	n.d.	[110,183]
	p.R56 *; p.327V>A	=	=	=	n.d.	n.d.	n.d.	n.d.	n.d.	n.d.	[185]
	p.R56 *; p.69R>C	=	=	=	↓	=	=	n.d.	n.d.	n.d.	[186]
	p.73R>C; p.368F>I	↓	=	=	=	=	=	=	=	=	[187]
	p.78S>G; p.144R>Q	=	=	=	n.d.	n.d.	n.d.	n.d.	n.d.	n.d.	[185]
	p.99P>L; p.99P>L	↓	↓	↓	↓	↓	↓	n.d.	n.d.	n.d.	[110,188,189]
	p.109R>W; p.109R>W	=	=	=	↓	=	↓	n.d.	n.d.	n.d.	[186]
	p.129G>R; p.129G>R	↓	n.d.	n.d.	↓	n.d.	n.d.	n.d.	n.d.	n.d.	[190,191]
	p.155R>P; p.353V>M	↓	n.d.	=	n.d.	n.d.	n.d.	n.d.	n.d.	n.d.	[188]
	p.183R>C; p.184R>C	↓	=	=	=	=	=	=	=	=	[187]
	p.184R>C; g1892A>G	↓	=	=	=	mild ↓	=	=	n.d.	↓	[110]
	p.184R>C; p.280L>F	n.d.	n.d.	n.d.	n.d.	n.d.	n.d.	n.d.	n.d.	n.d.	[192]
	p.277S>N; p.277S>N	↓	n.d.	=	n.d.	n.d.	n.d.	n.d.	n.d.	n.d.	[188]
	decreased levels BCS1L	↓	n.d.	↓	↓	=	=	n.d.	n.d.	n.d.	[193]
	mouse p.78S>G: p.78S>G	↓	=	=	↓	=	=	mild ↓	mild ↓	mild ↓	[115]
	mouse KO										[116]
***TTC19***	p.54P>A *	↓	n.d.	n.d.	n.d.	n.d.	n.d.	n.d.	n.d.	n.d.	[194]
	p.77Q>R *; p.77Q>R *	↓	n.d.	n.d.	=	n.d.	n.d.	n.d.	n.d.	n.d.	[195]
	p.173Q> *	↓	=	=	↓	n.d.	n.d.	n.d.	n.d.	n.d.	[196]
	p.185L>P	n.d.	n.d.	n.d.	=	n.d.	n.d.	n.d.	n.d.	n.d.	[197]
	p.186W> *; p.322G>M *	↓	n.d.	↓	n.d.	n.d.	n.d.	n.d.	n.d.	n.d.	[198]
	p.194R>N *	n.d.	n.d.	n.d.	n.d.	n.d.	n.d.	n.d.	n.d.	n.d.	[199]
	p.219L> *	↓	=	=	↓	n.d.	n.d.	n.d.	n.d.	n.d.	[196]
	p.261E>G *; p.261A>G *	n.d.	n.d.	n.d.	n.d.	n.d.	n.d.	n.d.	n.d.	n.d.	[200]
	p.277Q> *; p.277Q> *	n.d.	n.d.	n.d.	n.d.	n.d.	n.d.	n.d.	n.d.	n.d.	[201]
	p.313Q> *	↓	n.d.	n.d.	n.d.	n.d.	n.d.	n.d.	n.d.	n.d.	[202]
	p.321A> *; p.321A> *	↓	n.d.	n.d.	↓	n.d.	n.d.	n.d.	n.d.	↓	[114]
	p.324L>P	n.d.	n.d.	n.d.	=	n.d.	n.d.	n.d.	n.d.	n.d.	[197]
	mouse and human KO	↓	=	=	mild ↓	=	=	=	=	=	[203]
***UQCC2***	Protein absent	↓	↓	↓	↓	mild ↓	mild↑	↓	↓	↓	[85]
	p.[8R>P;10L>F];[8R>P;10L>F]	↓	↓	=	↓	↓	=	↓	↓	↓	[128]
***UQCC3***	p.20V>E; p.20V>E	↓	mild ↓	=	↓	↓	=	n.d.	n.d.	n.d.	[86]
***LYRM7***	p.13T>H *; p.13T>H *	↓	n.d.	n.d.	n.d.	n.d.	n.d.	n.d.	n.d.	n.d.	[204]
**LYRM7**	p.18R>D *; p.18R>A *	↓	mild ↓	mild ↓	↓	mild ↓	mild ↓	n.d.	n.d.	n.d.	[205]
	p.25D>N; p25D>N	↓	n.d.	n.d.	↓	n.d.	=	n.d.	n.d.	n.d.	[206]
	p.25D>N; p25D>N	↓	n.d.	n.d.	n.d.	n.d.	n.d.	n.d.	n.d.	n.d.	[204]
	p.72Q> *; p72Q> *	↓	n.d.	n.d.	n.d.	n.d.	n.d.	n.d.	n.d.	n.d.	[204]
	p.82K>N *; p.82K>N *	↓	n.d.	n.d.	↓	n.d.	n.d.	n.d.	n.d.	n.d.	[204]
	Protein absent	↓	n.d.	n.d.	n.d.	n.d.	n.d.	n.d.	n.d.	n.d.	[207]

*, a stop in the protein synthesis; ↓, decrease; ↑, increase; =, no change; n.d., not determined.
